# Identification of Genes Mediating *Drosophila* Follicle Cell Progenitor Differentiation by Screening for Modifiers of GAL4::UAS Variegation

**DOI:** 10.1534/g3.116.036038

**Published:** 2016-11-18

**Authors:** Ming-Chia Lee, Andrew D. Skora, Allan C. Spradling

**Affiliations:** Howard Hughes Medical Institute Research Laboratories, Department of Embryology, Carnegie Institution, Baltimore, Maryland 21218

**Keywords:** *Drosophila melanogaster*, *scny*, *tsh*, *cdc27*, *bora*, *mus312*, *Six4*, *Trl*, *mub*

## Abstract

The *Drosophila melanogaster* ovarian follicle cell lineage provides a powerful system for investigating how epigenetic changes contribute to differentiation. Downstream from an epithelial stem cell, follicle progenitors undergo nine mitotic cell cycles before transitioning to the endocycle and initiating differentiation. During their proliferative phase, follicle progenitors experience Lsd1-dependent changes in epigenetic stability that can be monitored using GAL4::UAS variegation. Eventually, follicle progenitors acquire competence to respond to Delta, a Notch ligand present in the environment, which signals them to cease division and initiate differentiation. The time required to acquire competence determines the duration of mitotic cycling and hence the final number of follicle cells. We carried out a screen for dominant modifiers of variegation spanning nearly 70% of *Drosophila* euchromatin to identify new genes influencing follicle progenitor epigenetic maturation. The eight genes found include chromatin modifiers, but also cell cycle regulators and transcription factors. Five of the modifier genes accelerate the acquisition of progenitor competence and reduce follicle cell number, however, the other three genes affect follicle cell number in an unexpected manner.

A regulated transition between progenitor proliferation and differentiation is critically important for tissue development and homeostasis. Downstream from stem cells, an appropriate level of progenitor proliferation is needed to generate enough cells to achieve normal organ size. Precocious differentiation leads to undersized tissues and may compromise tissue integrity, whereas delayed differentiation causes tissue overgrowth and possibly tumorigenesis. While cycling, progenitors display a flexible chromatin state (Iglesias-Bartolome *et al.* 2013), but epigenetic stability increases as cells differentiate. How progenitors control their proliferation and transit from a developmentally flexible to a developmentally restricted state remains poorly known.

The follicle cells of the *Drosophila* ovary provide an exceptionally favorable system for studying questions associated with epithelial progenitor growth and differentiation (Skora and Spradling 2010). Each developing ovarian follicle represents a highly reproducible system of cellular differentiation in miniature comprising somatic follicle cells, germline nurse cells, and an oocyte (Figure 1A). The 800 follicle cells on each mature follicle derive from two founder cells, each the daughter of a follicle cell stem cell (FSC). The two founders undergo five rounds of division (DIV1–5) before surrounding one oocyte and its 15 connected nurse cells to form a new follicle (King 1970; Margolis and Spradling 1995; Nystul and Spradling 2007). The follicle cell progenitors continue their amplification phase as a monolayer on the follicle surface with four more mitotic cycles (DIV6–9) before a major regulatory event, the mitotic/endocycle (M–E) transition, terminates proliferation and initiates differentiation (Deng *et al.* 2001; Sun and Deng 2005, 2007). Except for a few follicle progenitors that specialize early as polar or stalk cells (Margolis and Spradling 1995; Lopez-Schier and St Johnston 2001; Nystul and Spradling 2010), the progenitors now enter a differentiation phase and develop into multiple specialized follicle cell types that contribute to virtually every aspect of the egg’s internal structure and protective shell (reviewed in Wu *et al.* 2008; Klusza and Deng 2011).

**Figure 1:**
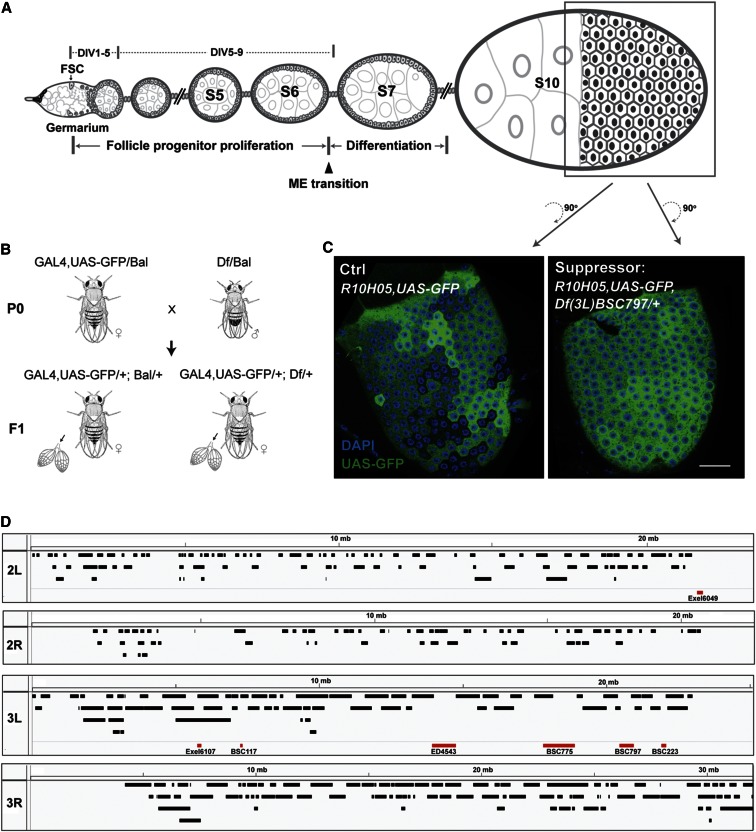
A deficiency screen to identify dominant modifiers of GAL4::UAS variegation in ovarian follicle cells. (A) The follicle cell lineage. A diagram of a developing string of *Drosophila* follicles (known as an ovariole) is diagrammed, showing the location of the follicle cell stem cell (FSC) midway in the germarium. After five divisions (DIV1–5), cells surround a cyst of 15 nurse cells and an oocyte to form a new follicle. Follicle cell progenitors continue to proliferate on the follicle surface (DIV5–9), until they undergo the mitosis–endocycle (ME) transition and begin to differentiate. Follicle stages such as stage 5 (S5) are indicated. Growth ceases at stage 10 (S10) and this stage was used to score GAL4:UAS variegation (arrows). (B) Crossing scheme used to identify GAL4::UAS modifiers. Deficiency lines heterozygous with a Balancer (Df/Bal) were individually crossed to one of three balanced GAL4::UAS-GFP stocks: (1) 179y-GAL4,UAS-GFP/FM7; (2) c768-GAL4,UAS-GFP/TM3; or (3) R10H05-GAL4,UAS-GFP. Female progeny (F1) from individual crosses were collected, fed wet yeast, and their ovaries were dissected 24–36 hr later, and stained with anti-GFP (green fluorescent protein) antibodies. Stage 10 follicles were mounted and GFP variegation patterns were compared between control (Bal/+) and heterozygous deficiency mutants (Df/+). (C) Example of a stage 10 follicle with a normal variegation pattern (Ctrl, left) and one in which variegation was suppressed by Df(3L)BSC797/+ (Suppressor, right). The presence of the suppressor is easily recognized by the more homogeneous GFP expression (reduced variegation). (D) Summary diagram of the deletions (boxes) present in tested lines from the deficiency kits on chromosomes 2L, 2R, 3L, and 3R. The seven deficiencies that scored positively as suppressors are highlighted in red. Scale bars, 20 μm. DAPI, 4’,6-diamidino-2-phenylindole.

Epigenetic changes within progenitors as they begin the process of differentiation have been extensively studied in cultured embryonic stem cells (Young 2011). Modifications to nucleosomal histones occur in concert with the establishment of heterochromatic zones, Polycomb-associated domains and active promoters. However, directly measuring chromatin changes in rare progenitor cells within a developing tissue is not usually technically feasible. Standard loss of function genetic screening is also challenging, since most genes involved in progenitor maturation are used widely and are essential. Recently, an alternative approach for finding genes involved in progenitor maturation was described for the *Drosophila* follicle cell lineage (Skora and Spradling 2010). The variegated GFP expression patterns of GAL4::UAS constructs were shown to report on progenitor epigenetic stability, and documented a steady increase in stability over the nine divisions. In early progenitors, the GFP expression level changes in one out of every 4–6 cells each division, whereas < 1 cell in 400 changes expression during the last division. Although the molecular mechanism of GFP variegation remains obscure, relevant cellular genes needed for epigenetic stabilization may nonetheless be identified by screening for variegation modifiers.

The usefulness of this approach was strongly supported when the first genes identified as variegation modifiers in follicle cell progenitors were studied (Lee and Spradling 2014). Reducing the gene dosage of *lysine-specific demethylase 1 (lsd1)* or the gene encoding its binding partner, *CoRest*, dramatically suppressed variegation. Variegation correlates with progenitor epigenetic instability, and progenitors must epigenetically stabilize to respond to the Notch signal that turns off the mitotic cell cycle (Lee and Spradling 2014). Consequently, suppressors of variegation such as *lsd1* or *CoREST* stabilize chromatin prematurely and have a reduced final number of follicle cells, while genes that counteract *lsd1*, such as *ash1* or *trx*, have an increased number (Lee and Spradling 2014). Thus, identifying additional variegation modifiers promises to provide an unbiased approach to discovering genes and genetic pathways that control progenitor growth and maturation.

We initially screened most of the *Drosophila* autosomes to identify chromosome regions whose dosage affects GAL4::UAS variegation and progenitor differentiation, using a collection of heterozygous deletions that include about 70% of euchromatic genes. Deletions that reproducibly modified variegation were studied further and genes located within their boundaries were scanned to identify single loci that were themselves dosage-sensitive. Using this strategy, we identified eight new genes, all as dominant variegation suppressors, and showed that they strongly influence epithelial progenitor differentiation. They include genes encoding chromatin modifiers [*Trithorax-like* (*Trl*) and *scrawny* (*scny*)], cell cycle regulators [*i.e.*, *mutagen-sensitive 312* (*mus312*), *aurora borealis* (*bora*), and *Cell division cycle 27* (*Cdc27*)], and transcription factors [*i.e.*, *teashirt* (*tsh*) and *Six4* (*Six4*)].

## Materials and Methods

### Drosophila stocks

Flies were reared on standard cornmeal agar yeast food at 22°. Fly strains used in this study were from Bloomington, VDRC, or members of the fly community as indicated. Oregon-R was used as control strain in this study. 179y-Gal4, c768-Gal4, and R10H05-Gal4 (Jenett *et al.* 2012) were described previously (Skora and Spradling 2010). The deficiency stocks used were obtained from the Bloomington *Drosophila* Stock Center (BSC) as part of the Exelixis deficiency kit (Parks *et al.* 2004) or the BSC deficiency kit (Cook *et al.* 2012). Nearly all these deficiencies were generated from sequenced insertions on a common genetic background, hence their presumed endpoints are molecularly defined. At the present time, most stocks have been genetically tested and results from lines that have yet to be verified are not shown (all were negative). RNAi lines were obtained from the Harvard RNAi project (Perkins *et al.* 2015) and from the Vienna *Drosophila* Stock Center (VDRC) (Dietzl *et al.* 2007).

### Epigenetic plasticity assay

Candidate gene mutations were combined with one or more GAL4 lines, R10H05-Gal4, c768-Gal4, or 179y-Gal4. R10H05 drives GFP expression beginning in FSCs, while c768 and 179y only initiate GFP expression after the M–E transition. All three Gal4 lines showed indistinguishable GFP variegation patterns at stage 10 (Skora and Spradling 2010) and line-specific behavior was not observed in these experiments.

To quantitatively measure epigenetic plasticity during the final five follicle cell divisions, we calculated the frequency with which changes in GFP expression took place at each division in GAL4::UAS-GFP variegating follicles (Skora and Spradling 2010). Briefly, using stained and mounted stage 10B follicles, we measured the postmitotic sizes of individual GFP patches (known to be clonal in origin) indicating the division at which particular events occurred during progenitor growth (by rounding to the nearest power of two). For instance, one-cell clones derive from the last mitotic division (nineth division) while two-cell clones originate at DIV8. By measuring the size of every GFP patch within a known amount of follicular surface (about 250 cells/follicle scored), the number of epigenetic changes that produced an expression level change could be determined during each division from DIV5–9 for a known total number of scored follicle cells. Then, by dividing the number of such epigenetic events by the total number of divisions required to produce the scored cells, the probability of an epigenetic change at each division could be calculated (“change probability”). From the change probability profiles we calculated the modification index (MI), as described in the text.

### Immunostaining and microscopy

Ovary staining and visualization was carried out as described previously (Lee and Spradling 2014). Ovaries were dissected in Grace’s solution. Dissected ovaries were fixed in 3.7% formaldehyde in 1 × PBS for 15 min at room temperature. Primary antibodies were used overnight at 4°. Antibodies and dilutions used in this study are rabbit anti-GFP (Invitrogen, 1:1000). Secondary antibodies from Invitrogen were goat anti-rabbit 488. Stained ovaries were mounted in Vectashield on glass slides. Images were taken on a Sp5 confocal microscope and processed with ImageJ or Metamorph software.

### Data availability

The authors state that all data necessary for confirming the conclusions presented in the article are fully represented within the Tables and Figures.

## Results

### Screening deletions spanning most of the second and the third chromosome

The general strategy for identifying genomic regions that are dosage sensitive for GAL4::UAS variegation is shown in Figure 1, B and C. We used two collections of largely isogenic strains bearing balanced heterozygous molecularly characterized deficiencies (also known as deletions). Chromosome 2 stocks were from the Exelixis collection as it existed in 2009 (Parks *et al.* 2004). Chromosome 3 stocks were from the “the deficiency kit” circa 2013, which was generated and characterized by the Bloomington *Drosophila* Stock Center (Cook *et al.* 2012), and which are mostly on the same genetic background as the Exelixis stocks. Subsequent studies (Cook *et al.* 2012; FlyBase 2016) confirmed that ∼70% of the Exelixis chromosome 2 stocks carried a deletion in the indicated region; only results with these deficiencies are reported.

Each strain containing a balanced deletion was crossed to either c768-GAL4 or c179-GAL4, and the GAL4::UAS-GFP variegation patterns in ovarian follicles were compared between progeny carrying or lacking the deficiency (Figure 1B). Female progeny either containing or lacking the deletion in question, as well as the GAL4::UAS-GFP constructs, were selected, their ovaries were stained with anti-GFP antibodies, and stage 10 follicles were scored to determine if the level of GFP variegation in follicle cells was normal, suppressed, or enhanced (Figure 1C).

Ovaries from control females, and ovaries from females bearing most deletions, showed a normal pattern of GFP variegation that has been extensively characterized previously (Skora and Spradling 2010; Lee and Spradling 2014). Candidate deficiencies that appeared to modify variegation in the initial screen were recrossed to the same and other GAL4::UAS-GFP tester strains and larger numbers of females were dissected and follicles scored. These tests identified seven deficiencies that behaved consistently as suppressors of variegation (Figure 1D). The identified regions are heterogeneous in size and contain 10 to over 100 genes (Table 1). No regions enhancing variegation were observed.

**Table 1:** Summary of variegation modifier screen using Bloomington deficiencies

Arm	Df Lines Tested (*N*)	% Coverage (Euchromatin)	Df Positive (*N*)	Names
2L	103	85	1	Exel6049
2R	67	70	0	
3L	71	90	6	Exel6107, Df(3L)ED4543, Df(3L)BSC117, Df(3L)BSC223, Df(3L)BSC775, Df(3L)BSC797
3R	98	95	0	

For each autosomal chromosome arm, the number of deficiency lines tested and the names and number that scored positive as modifiers (suppressors) of GAL4::UAS variegation are listed. Df, deficiency.

### Identification of a specific gene within Df(3L)ED4543

We continued our search for genes needed in two doses for normal follicle progenitor differentiation within the positive deletions on the assumption that one or at most two genes were responsible for the dosage effect of the entire deletion. Thus, for each of the positive deletions we obtained smaller deletions, when available, that further subdivide the region of interest. In addition, we obtained mutations (null if possible), or RNAi lines targeting genes within the region, in order to identify individual genes responsible for modifying variegation.

**Figure 2:**
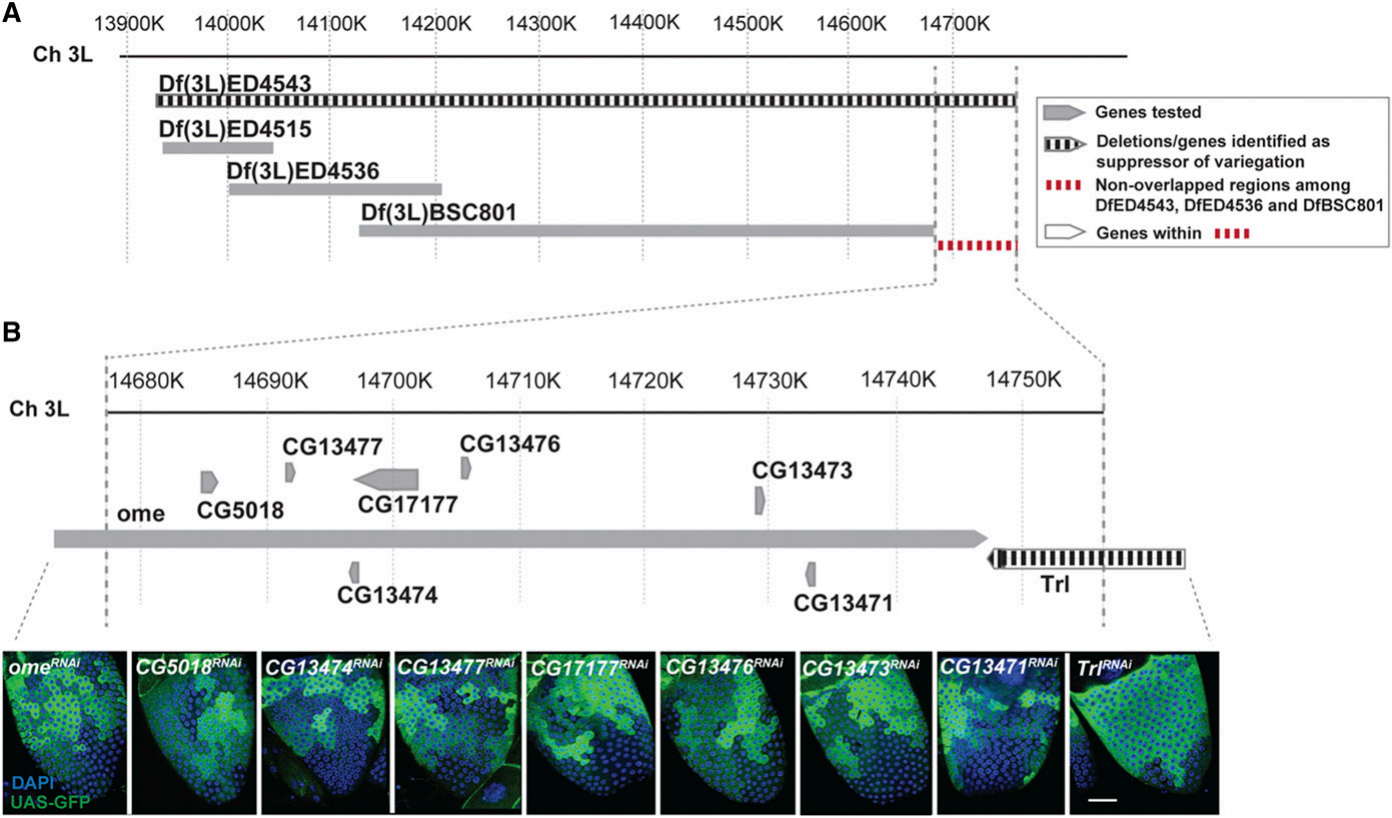
Mapping a variegation suppressor in Df(3L)ED4543 to *Trl*. (A) A diagram of the genomic region containing Df(3L)ED4543 with nucleotide coordinates shown at the top. The extent of the deletions in Df(3L)ED4543, Df(3L)ED4515, Df(3L)ED4536, and Df(3L)BSC801 are show as boxes. The color and shading of the boxes indicates whether variegation was suppressed [vertical bars, Df(3L)ED4543] or wild-type [plain gray, Df(3L)ED4515, Df(3L)ED4536, and Df(3L)BSC801]. The red hatched region indicates the remaining portion of Df(3L)ED4543 in which the suppressor might be located. (B) The region containing the suppressor shown on an expanded scale (see DNA coordinates above). The boxed arrows below the line are genes, which were tested individually for their ability to suppress GFP variegation using R10H05GAL4 in 20–40 stage 10 follicles each. All the genes failed to suppress (gray shading) except *Trl* (black vertical bars). Representative images of the GFP expression in a stage 10 follicle of each genotype (as indicated) is shown at the bottom. Note the much more uniform GFP expression in the follicle from *Trl*[*RNAi*]. DAPI (blue). Scale bars, 20 μm. Ch, chromosome; DAPI, 4’,6-diamidino-2-phenylindole; GFP, green fluorescent protein.

The results of these studies are summarized in Figure 2 in the case of the deletion Df(3L)ED4543, which removes DNA from cytogenetic position 70C6-70F4 on chromosome 3L, a region of 822.8 kb between coordinates 13,935,225–14,758,040 (R6) that contains 78 genes. Three smaller deletions—Df(3L)ED4515, Df(3L)ED4536, and Df(3L)BSC801—that subdivide Df(3L)ED4543, allowed the candidate suppressor to be localized to a smaller region (Figure 2). All three deletions showed normal variegation levels (Table 2), leaving only a small interval at the proximal end of the original deletion containing nine genes to be tested further.

**Table 2:** Deficiencies used

No.	Name	Phenotype	Genes
1	Exel6049	Suppressor	23
2	Exel6107	Suppressor	34
3	Df(3L)ED4543	Suppressor	78
3A	Df(3L)ED4515	Wt	18
3B	Df(3L)ED4536	Wt	24
3C	Df(3L)BSC801	Wt	44
4	Df(3L)BSC117	Suppressor	10
5	Df(3L)BSC223	Suppressor	22
6	Df(3L)BSC797	Suppressor	68
6A	Df(3L)BSC449	Wt	48
6B	Df(3L)BSC452	Suppressor	27
6C	Df(3L)BSC796	Wt	43
7	Df(3L)BSC775	Suppressor	144

The names and modifier phenotypes of the deficiencies that scored positively in the initial round, as well as additional deficiencies that subdivide them are given. The number of annotated genes in each deficiency is shown. No. number; Wt, wild-type.

Null mutations of all nine genes were not available, but strains targeting each gene for knockdown via RNAi were obtained (*Materials and Methods*). For RNAi to be effective, we used a driver line, R10H05-GAL4 (Jenett *et al.* 2012), that expresses throughout the entire period of follicle progenitor development (Skora and Spradling 2010). Although the exact level of knockdown of gene expression using RNAi is likely to vary, due both to driver variegation and the variable efficiency of individual RNAi lines, we observed previously that RNAi lines targeting *Lsd1* or *CoREST* modified variegation in the same manner as a heterozygous mutation in the genes (Lee and Spradling 2014).

Therefore, to further map the suppressor locus in Df(3L)ED4543, RNAi lines recognizing each gene were crossed to the R10H05-GAL4 driver and progeny females were selected in which R10H05-GAL4 was present along with both the UAS-GFP and UAS-RNAi constructs. Females inheriting the driver, but which lacked UAS-RNAi, served as controls. Representative stage10 follicles are shown for each of the nine genes in the relevant interval (Figure 2B). The results were highly consistent with the assumption that a single gene in this region is responsible for suppressing GAL4::UAS variegation. RNAi lines targeting eight of the genes showed no changes in the pattern of variegation that could be recognized by eye. In contrast, the ninth gene, Trithorax-like, strongly suppressed variegation, drastically reducing the number of epigenetic clones with different levels of GFP expression visible per follicle.

### Identification of two specific genes within Df(3L)BSC117

**Figure 3:**
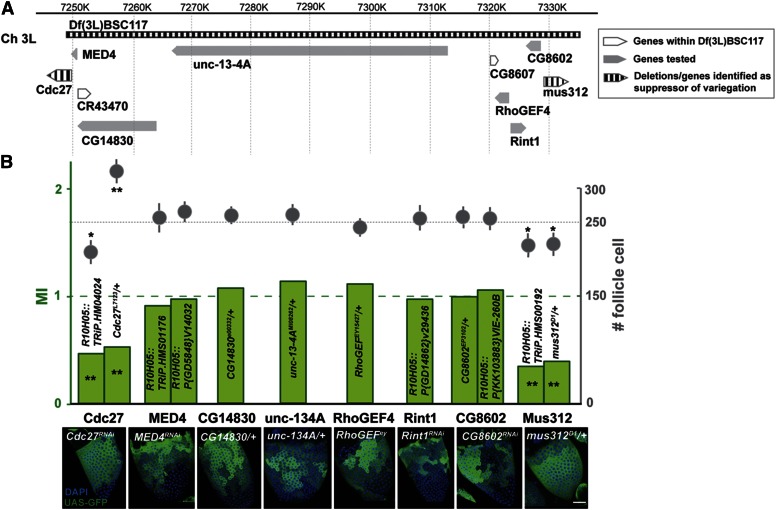
Mapping *Cdc12* and *mus312* as variegation suppressors in Df(3L)BSC117. (A) The genomic region surrounding Df(3L)BSC117 is shown, including the deletion, and the position of the 10 genes are shown as boxed arrows. Labeling conventions are the same as in Figure 2 and are described in the key. Two genes were not tested (white boxes). (B) Two genes, *Cdc27* and *mus312*, suppressed R10H05-GAL4::UAS-GFP variegation based on stage 10 GFP patterns (shown below), and confirmed by two other measurements. A bar graph (*y*-axis at left) indicates the mean MI (see *Materials and Methods*) for the indicated alleles. Above, the number of follicle cells per mounted stage 10 follicle (mean ± SD) in the mutant or RNAi-bearing heterozygotes is plotted (see *y*-axis at right). Two follicle cell plots or two boxes per gene indicate that two alleles were tested as labeled. Scale bars: 20 μm. * *P* < 0.05; ** *P* < 0.01; Student’s *t*-test. Ch, chromosome; DAPI, 4’,6-diamidino-2-phenylindole; GFP, green fluorescent protein; MI, modification index; RNAi, RNA interference.

A similar approach was taken to identify genes responsible for the strong variegation suppression of Df(3L)BSC117. Df(3L)BSC117, a smaller deletion than Df(3L)ED4543, removes cytogenetic region 64D6-64E7, an 85.5 kb domain corresponding to coordinates 7249475–7334986 (R6) housing nine protein-coding genes and one putative noncoding RNA (Figure 3A). Here, a combination of heterozygous mutations or RNAi disruption was used to test the effects of eight genes on GAL4::UAS-GFP variegation. Based on the variegation patterns, six of the eight tested loci scored no differently than wild-type. One very small gene, CG8607, and the candidate noncoding RNA, CR43470, could not be tested. However, interestingly, strong suppressor activity was observed for two genes, *cdc27* and *mus312* (Figure 3B). Both these genes scored positively using two different alleles.

### Identification of five additional suppressors

The same approach as described above for Df(3L)ED4543 and Df(3)BSC117 was carried out for the remaining five deletions that had scored as variegation suppressors. Overlapping deficiencies (Table 2), mutant stocks, and RNAi lines were obtained for as many of the genes located within these regions as possible. Variegation tests were carried out as described above, and one gene that scored as a suppressor in each deficiency was eventually identified. Because of the statistical expectation that only one gene would score positively in most deletions, genes that had not yet been tested after the positive gene was confirmed were usually not pursued further.

### Validation of the identified modifiers

To further validate the mapping and identification of variegation modifier genes identified so far, we performed additional tests. All eight genes are expressed in stage eight follicles by RNAseq (Sieber and Spradling 2015). For each of the eight genes, we identified two or more independent alleles, or RNAi disruption lines, and quantitatively analyzed their effects on variegation (see *Materials and Methods*). A suppressor would reduce the probability that GFP expression levels would change (the “change probability”) over late divisions, whereas an enhancer would increase the change probability. Consequently, we defined a MI as the sum over divisions five to nine of the change probabilities of variegating follicles from mutant females divided by the same quantity from control follicles. If there is no modification of variegation, then MI = 1. However, suppressors that reduce the change probabilities will have MI < 1, while enhancers will increase change probabilities and score with MI > 1.

We tested this approach for the genes within Df(3L)BSC117, and found that expectations based on visual scoring were validated (Figure 3B). The MI values of all six genes that did not visibly modify variegation were around 1. In contrast, the two genes scoring as positive based on variegation pattern were confirmed as suppressors since their MI values were reduced to ≤ 0.5 in each to two alleles tested.

We also tested the genes using an assay that is not directly related to variegation by measuring the mean number of follicle cells present in photographs of stage 10 follicles. Previous studies of *lsd1* and its interacting genes revealed that there is a strong biological connection between follicle progenitor variegation levels and the timing of the M–E transition, which in turn controls follicle cell number (Lee and Spradling 2014). Variegation correlates with progenitor epigenetic instability, and progenitors must epigenetically stabilize to activate the Notch signal that turns off the mitotic cell cycle. Consequently, suppressors of variegation, such as *lsd1* or *CoREST*, stabilize prematurely and have a reduced final number of follicle cells while genes that counteract *lsd1*, such as *ash1* or *trx*, have an increased number.

The genes in Df(3L)BSC117 mostly validated this connection (Figure 3B). All six genes that did not affect variegation had stage10 follicles with about 250 follicle cells per field (251 ± 5). In contrast, the number of follicle cells in *mus312* disruptions was only 218 ± 7, or 220 ± 7, which is significantly less (*P* < 0.0001 *t*-test). *Cdc27*[*TRiP .HM04024*] follicles also contained significantly fewer follicle cells (215 ± 6; *P* < 0.001 *t*-test). However, unexpectedly, *Cdc27*[*L7123*] had an increased number of follicle cells per side, 324 ± 7 (Table 3). This result suggests that, even though follicle progenitor epigenetic stability is accelerated, *Cdc27*[*L7123*] delays the follicle cell M–E transition, possibly due to a downstream function of Cdc27 in the onset of the endocycle. The transposon insertion in the L7123 allele may cause a regulatory change that differs in effect from the RNAi knockdown allele.

**Table 3:** Modifier genes and their properties

Gene	Symbol	Location	Allele	ΣPc_d5-9_ Mean ± SD (*N*)	MI ΣPc_d5-9_^m/+^/ΣPc_d5-9_^WT^	# Cells Mean ± SD (*N*)	Human Orthologs
Control				0.35 ± 0.15 (88)	1	251 ± 5 (44)	No
*teashirt*	*tsh*	2L:21,828,593..21,837,011	8	0.08 ± 0.08 (20)	0.22	**281 ± 11** (20)	TSHZ3
			2757R8	0.16 ± 0.08 (21)	0.46	**269 ± 6** (21)	
*scrawny*	*scny*	3L:5,769,002..5,776,271	20331	0.04 ± 0.06 (19)	0.11	*227 ± 5* (19)	USP3
			TRiP. HMS02044	0.12 ± 0.06 (21)	0.36	260 ± 5 (21)	
*aurora borealis*	*bora*	3L:18,622,692..18,624,751	EY21751	0.15 ± 0.14 (15)	0.42	*225 ± 8* (15)	BORA
*Trithorax-like*	*Trl*	3L:14,747,929..14,761,049	s2325	0.14 ± 0.10 (24)	0.41	*196 ± 8* (24)	NACC1
			MI02741	0.14 ± 0.15 (19)	0.40	*233 ± 4* (19)	
*Mutagen sensitive 312*	*mus312*	3L:7,329,132..7,333,471	D1	0.07 ± 0.11 (16)	0.19	*218 ± 7* (16)	SLX4
			TRiP. HMS00192	0.11 ± 0.06 (16)	0.31	*220 ± 7* (16)	
*Cell division cycle 27*	*Cdc27*	3L:7,245,481..7,249,340	L7123	0.13 ± 0.10 (15)	0.38	**324 ± 7** (15)	CDC27
			TRiP. HM04024	0.12 ± 0.13 (21)	0.36	215 ± 6 (21)	
*Six4*	*Six4*	3L:20,788,886..20,792,777	EY09833	0.18 ± 0.08 (19)	0.52	254 ± 8 (19)	SIX4
			TRiP. HM05254	0.18 ± 0.15 (17)	0.50	245 ± 10 (17)	
*mushroom body-expressed*	*mub*	3L:21,844,788..21,932,702	MI10575	0.13 ± 0.16 (16)	0.38	*217 ± 7* (16)	PCBP1-3
			TRiP. HMS00189	0.13 ± 0.08 (16)	0.37	*220 ± 6* (16)	

The name, location, allele, and modifier properties of one or two alleles of the eight suppressor of variegation genes are summarized. ΣPc_d5-9_ = the sum of the change probabilities during DIV5–9 [mean ± SD (*N* = number of follicles scored)]. MI = ΣPc_d5-9_^m/+^/ΣPc_d5-9_^WT^. All ΣPc_d5-9_ values (and hence MI values) differed significantly between modifier alleles and control (*P* < 0.001). #Cells = the number [mean ± SD (*N* = number of follicles scored)] of follicle cells on the visible surface of a mounted stage 10 follicle. Values shown in italics (decrease) or bold (increase) differ significantly from control (*P* < 0.01, Student’s *t*-test). MI, modification index; TRiP, transgenic RNAi Project.

We went on to measure the MI index and mean follicle cell number for two alleles of all the modifier genes identified in this screen except for *bora* (Table 3). As expected, all eight suppressors identified showed a sum of DIV5–9 change probabilities that differs from controls (= MI) by more than four SD (*P* < 0.001, Table 3). Moreover, five of the eight suppressors, and one allele of the sixth, *Cdc27^L7123^*, caused follicles to contain fewer follicle cells (Table 3), as expected if accelerated epigenetic maturation moves the M–E transition earlier, like in *Lsd1* and *CoREST* heterozygotes (Lee and Spradling 2014). Interestingly, these experiments provided the first evidence that epigenetic stability, as measured by variegation and follicle cell number, can be separated. *Six4* suppressed variegation, but did not significantly alter the number of follicle cells. *tsh* alleles suppressed variegation but increased the total number of follicle cells (Table 3). Finally, *Cdc27*[*L7123*] increased follicle cell number, but *Cdc27*[*TriP.HM00424*] reduced it. A plausible explanation for the separation of follicle cell number and variegation is that some genes have a second function in actually carrying out the mitotic endocycle switch after progenitor chromatin has become modified. Cdc27 is a conserved component of the APC/C E3 ubiquitin ligase that orchestrates the cell cycle metaphase to anaphase transition as well as mitotic exit. It seems plausible that altering *Cdc27* dosage could extend mitotic cycling longer than normal.

## Discussion

### Identifying new genes that modify GAL4::UAS variegation and affect follicle cell progenitors

Our results document the value of screening for GAL4::UAS modifiers in *Drosophila* follicle cells to identify genes involved in progenitor differentiation. A previous candidate-based study identified *lsd1*, *lid*, and *CoRest*, as variegation suppressors and as repressors of follicle progenitor differentiation (Lee and Spradling 2014). Conversely, *trx* and *ash1* were documented as variegation enhancers. The known function of these genes in demethylating or methylating histone H3K4 suggested that removal of H3K4me marks at one or more target promoters, possibly Notch target genes, represses a premature M–E transition in developing progenitors. A developmentally regulated decline in Lsd1 level was observed during progenitor development and was proposed to time differentiation onset (Lee and Spradling 2014).

The two new genes identified in this screen that encode chromatin modifiers support this model and suggest that the targets lie within Polycomb domains. *Scny* encodes a ubiquitin protease whose deubiquitinylation of histone H2B is likely to suppress the acquisition of H3K4me3 at Polycomb-repressed promoters (Sun and Allis 2002; Bray *et al.* 2005; Buszczak *et al.* 2009). The other chromatin modifier we identified is *Trl*, which encodes the GAGA factor and positively regulates some genes, but which also functions in Polycomb group-dependent silencing (Mishra *et al.* 2003; Berger and Dubreucq 2012). Consequently, Trl, like Lsd1, CoREST, and Scny, functions in epigenetic processes that take place at promoters to restrain gene activation.

The variegation screen also identified a new class of modifier, genes affecting the cell cycle including *mus312*, *Bora*, and *cdc27*. Follicle progenitors display a relatively constant 11 hr cell cycle, but the length of S phase increases significantly during the cycles leading up to the M–E transition (Skora and Spradling 2010). Mus312 functions in DNA repair (Andersen *et al.* 2009) and may be required for normal cycling and S-phase length expansion in follicle progenitors. Bora partners with Aurora A to activate Polo at centrosomes late in G2, promoting entry into M phase (Seki *et al.* 2008). In contrast, Cdc27 is a component of the APC/C E3 ubiquitin ligase complex that interacts with mitotic checkpoints and regulates mitotic exit. The fact that Mus312, *bora*, and *cdc27* suppress variegation in follicle progenitors suggests that the nature of progenitor cell cycles plays an important role in restraining premature differentiation. The target genes, whose expression is repressed by chromatin modifiers, may also be restrained from premature activity by their time of replication, and this property may be altered by changes in the dosage of Mus312, *bora*, or *cdc27*.

Another one of the modifier genes, *mushroom body defective* (*mub*), encodes a gene regulatory protein that may influence the cell cycle. Mub is a poly-C binding protein likely involved as an RNA-binding protein (Stoiber *et al.* 2015) in posttranscriptional gene regulation, especially of splicing (Brooks *et al.* 2015). One of its three polyC-binding, human orthologs, PCBP1, has been implicated in the translational regulation of *cdc27* (Link *et al.* 2016).

Finally, the screen identified two genes, *Six4* and *teashirt*, encoding known transcription factors. Six4 helps pattern cell identities in the embryonic mesoderm (Clark *et al.* 2006), the tissue of origin of FSCs and follicle progenitors. These genes may control the expression levels of other variegation modifiers. In addition, both Six4 and Tsh are members of the “retinal determination network (RDN),” a collection of genes whose roles were originally characterized in studies of *Drosophila* eye development (Datta *et al.* 2011) but which is now known to control cell specification and cell proliferation in multiple developing epithelia in *Drosophila* and mammals (Kong *et al.* 2016). Thus, changes in the expression of *tsh* or *Six4* in follicle progenitors either directly or indirectly affect progenitor proliferation and differentiation. These findings argue that other RDN members that are known to be expressed in follicle progenitors, such as *Eyes absent* (*Eya*), should be tested as modifiers.

### Some variegation suppressors do not decrease follicle cell number

All genes encoding chromatin modifiers that suppress variegation decrease the number of follicle cells per follicle, while chromatin modifier genes that enhance variegation increase this parameter (Table 3; Lee and Spradling 2014). This is expected if chromatin modifier genes alter the timing of the M–E transition. Unexpectedly, this study identified three variegation suppressor genes that either increase the number of follicle cells (tsh, Cdc27) or leave it unchanged (Six4). Thus, enhancing the speed of the epigenetic stabilization reported by GAL4::UAS-GFP variegation is not sufficient to accelerate the M–E transition. The transcription factors (tsh and Six4) may reduce expression of chromatin modifiers and thereby suppress variegation, but independently alter expression of other target genes that normalize or even delay the M–E transition. Cdc27 may suppress variegation by altering the cell cycle or the rate of turnover of other relevant proteins in a manner that partially uncouples epigenetic stabilization for mitotic exit.

### Comparison to position effect variegation (PEV) modifier screens

Our approach was modeled after the strategy of screening for modifiers of (PEV), which has proven to be a successful strategy for identifying genes that regulate gene silencing (Gowan and Gay 1934; Reuter and Wolff 1981; Sinclair *et al.* 1983; review: Elgin and Reuter 2013). PEV resembles GAL4::UAS variegation, in that both are unexplained unstable genetic effects that only result from abnormal genotypes. It is encouraging to recall that identifying a full repertoire of suppressors and enhancers of variegation [*i.e.*, Su(var) and E(var), respectively] required multiple genetic screens over many decades (Elgin and Reuter 2013). These two types of variegation are distinct (Skora and Spradling 2010), but there is some overlap among modifier genes. For example, *Trl* and *Lsd1* were both previously identified as PEV suppressors, but most modifiers of these two processes are distinct.

### Screen specificity and sensitivity

**Figure 4:**
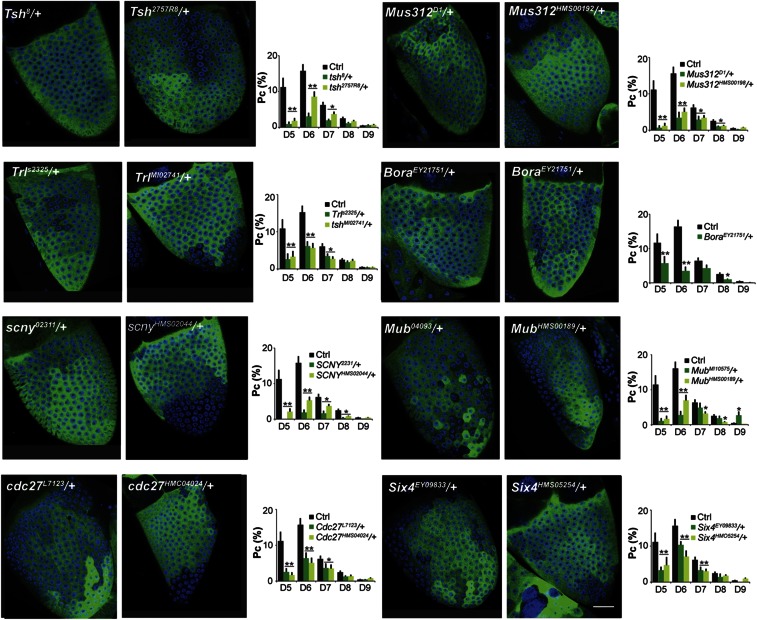
Effect on variegation of the eight identified suppressors. The Pc profiles of two alleles (green bars) of each of the eight identified variegation suppressors are shown, along with a representative picture of a stage 10 follicle from each suppressed allele (or RNAi line). These studies all used R10H05-GAL4. For control stage 10 pictures, see Figure 1C (Ctrl). Pc (%) represents the percentage of time a GAL4 level changed at the division (D) indicated on the *x*-axis. Control (Ctrl, black) represents the change profile of wild-type follicles. Columns represent the mean ± SEM (error bar). For each RNAi/mutant line, the change probability was calculated from at least two independent experiments and more than 20 follicles were scored for each experiment. Common control values for R10H05 were based on 88 scored follicles. Scale bar for all pictures: 20 μm. PC, change probability; RNAi, RNA interference.

All genetic screens are tradeoffs between sensitivity and specificity. Based on the characteristics of the eight new modifier genes, the current screen appears to have been highly specific. All eight genes are all expressed in stage 7–8 follicles (Sieber and Spradling 2015). As expected for a fundamental process like progenitor differentiation, all have strong human homologs (Figure 4), and for several their function is already connected to known processes important to differentiating follicle progenitors. Moreover, these modifiers do not just affect the expression of constructs, but alter the timing of progenitor differentiation, leading to changes in the number of follicle cells.

There are additional indications, however, that the screen was not very sensitive and missed many other genes that also participate in progenitor development. First, the number of modifiers, only eight in a screen of ∼70% of *Drosophila* euchromatin, seems far too low. In addition, only suppressors but no enhancers were identified. The genome coverage appeared to be nonrandom, with seven of eight genes coming from one chromosome arm. It is known that Hippo signaling plays a role in the ME transition (Polesello and Tapon 2007) in addition to Notch signaling (Deng *et al.* 2001), yet no genes in these pathways were uncovered as modifiers. Tellingly, deletions spanning suppressor genes that were later documented by candidate screening, including *lsd1*, *CoREST*, *lid*, *trx*, and *ash1*, were not scored as unambiguously positive in the initial screen. Some of these limitations were caused by the incomplete coverage of molecularly defined, validated chromosome 2 deficiencies when these experiments began in 2009.

There are several other likely reasons the screen showed relatively low sensitivity. It takes a strong suppressor to generate a readily visible difference in variegation pattern that can be recognized by eye without more detailed analysis. We suspect that it is easier to detect suppressors that make a variegated pattern appear quite uniform, compared to enhancers that make an already variegated pattern appear even more variegated. The change probability graphs in Figure 4 show that Div5 and Div6 are the critical divisions for detection as a suppressor. Suppressors acting only early or later in progenitor development might have been missed. Other problems inherent to the use of deficiencies as the initial screen are the possibility of genetic interactions occurring within individual deletions and the inability to identify genes as dominant modifiers whose effects are not dose sensitive. Consequently, all negative results from this screen require further verification.

### Potential improvements

Based on these observations, several changes in strategy would likely allow more sensitive screening to be carried out and a wider range of modifiers to be identified. The first improvement would be to carry out the modifier screen on an already suppressed background, such as an *lsd1* heterozygote. While we identified one Trithorax group gene here, *Trl*, two other genes, *trx* and *ash1*, were shown previously to affect progenitor differentiation, but they only modified variegation in a readily detectable manner by reversing suppression (Lee and Spradling 2014). *Lsd1/+* likely represents a “sensitized background” for detection of genes that promote differentiation and enhance variegation. Another strategy would be to focus on the smallest clones, those of 1–2 cells, which are normally rare. Genes that enhanced the number of these small clones might be detectable, even if the overall pattern of variegation, which is dominated by events occurring at DIV5 and DIV6, was not clearly changed. Finally, it was encouraging that RNAi using the R10H05 driver was often highly effective in modifying variegation. It would be worth screening many other candidate genes using RNAi, or even unselected genes using this approach.

A future application of modifier screening with great potential will be to identify relevant noncoding RNAs. Evidence is growing that long noncoding RNAs contribute to the composition and localization of chromatin-modifying complexes (Tsai *et al.* 2010; Fatica and Bozzoni 2014). Indeed, some of these complexes are known to include proteins, such as Lsd1 and CoREST, that have already been implicated as GAL4::UAS modifiers. Consequently, there is reason for optimism that GAL4::UAS variegation can be used as a platform for identifying not just genes encoding proteins but also RNAs that modulate epigenetic plasticity and inheritance during differentiation.
